# Exploring the Hidden World of Vectors of Chagas Disease: A Fascinating Look at the Taxonomic Aspects of the *Psammolestes* Genus (Hemiptera, Triatominae)

**DOI:** 10.3390/life13051081

**Published:** 2023-04-25

**Authors:** Jader de Oliveira, Kaio Cesar Chaboli Alevi, Carlos Eduardo Almeida, Nicoly Olaia, Gustavo Lázari Cacini, Cleber Galvão, Heitor Miraglia Herrera, Filipe Martins Santos, João Aristeu da Rosa

**Affiliations:** 1 Laboratório de Entomologia em Saúde Pública, Departamento de Epidemiologia, Faculdade de Saúde Pública, Universidade de São Paulo, Av. Dr. Arnaldo 715, São Paulo 01246-904, SP, Brazil; 2 Laboratório Nacional e Internacional de Referência em Taxonomia de Triatomíneos, Instituto Oswaldo Cruz (FIOCRUZ), Av. Brasil 4365, Pavilhão Rocha Lima, sala 505, Rio de Janeiro 21040-360, RJ, Brazil; 3 Instituto de Biologia, Departamento de Zoologia, Universidade Federal do Rio de Janeiro—UFRJ, Rio de Janeiro 21941-902, RJ, Brazil; 4 Faculdade de Ciências Farmacêuticas, Universidade Estadual Paulista (Unesp), Rodovia Araraquara-Jaú km 1, Araraquara 14801-902, SP, Brazil; 5 Laboratório de Biologia Parasitária, Ciências Ambientais e Sustentabilidade Agrícola, Universidade Católica Dom Bosco, Campo Grande 79117-010, MS, Brazil

**Keywords:** Rhodniini, *Psammolestes tertius*, *Psammolestes coreodes*, *Psammolestes arthuri*, chagas disease vectors

## Abstract

Chagas disease (CD) is a neglected illness affecting approximately seven million individuals, with vector transmission occurring via triatomine bugs. The Rhodniini tribe comprises 24 species, grouped into the *Rhodnius* and *Psammolestes* genera. Given the importance of accurately identifying CD vectors, the taxonomy of *Psammolestes* spp. was revisited using morphological and morphometric data. Specimens of *P. tertius*, *P. coreodes*, and *P. arthuri* were collected, and the morphological characteristics of the head, thorax, abdomen, and eggs were analyzed. Morphometric studies of eggs were also conducted. Dichotomous keys allowing for the differentiation of *Psammolestes* spp. were elaborated based on adult insect and egg morphological characteristics. Through these studies, it was possible to differentiate the three *Psammolestes* species and confirm that this genus should not be classified under the *Rhodnius* genus, contributing to Rhodniini taxonomy.

## 1. Introduction

Chagas disease (CD) is a neglected disease that affects approximately eight million people and places another 25 million at risk of infection [1], resulting in about 30,000 new cases of infection and 14,000 deaths from Chagas complications every year [2]. This disease is caused by the protozoan *Trypanosoma cruzi* (Chagas, 1909) (Kinetoplastida, Trypanosomatidae) and can be transmitted in several ways [1]. However, vector transmission by triatomines is considered the main form of dissemination of CD in Latin America [1].

The vector transmission of CD occurs by triatomines [1]. These hematophagous insects have the habit of defecating during hematophagy [1,3]. If they are infected by *T. cruzi*, they release the parasite in the feces/urine, causing the infection of the host [1,3]. Another form of infection also associated with infected triatomines is oral transmission, in which triatomines, or feces/urine, can be processed together with fruit juices, causing contamination of food [4].

Composed of 158 species and 18 genera, the subfamily Triatominae (Hemiptera and Reduviidae) is divided into five tribes: Alberproseniini, Bolboderini, Cavernicolini, Rhodiniini and Triatomini [5,6,7]. The last two tribes represent the largest number of taxa and give the species a great epidemiological importance [5,6,7].

The Rhodniini tribe is composed of 24 species grouped in the *Rhodnius* Stål, 1859, and *Psammolestes* Bergroth, 1911, genera [5]. The genus *Psammolestes* was described by Bergroth in 1911 from the description of *P. coreodes* Bergroth [8]. After 15 years, Cesar Pinto [9] described *Eutriatoma arthuri* Pinto, 1926, which was later transferred to the genus *Psammolestes* by Del Ponte [10]. In 1965, the third and last species grouped in the genus *Psammolestes* was described as *P. tertius* by Lent and Jurberg [11].

*Psammolestes* spp. are endemic species in South America; *P. arthuri* was reported in Colombia and Venezuela [12], *P. coreodes* was reported in Argentina, Bolivia, Brazil and Paraguay [12,13], and *P. tertius* was reported in Brazil and Peru [12,14,15]. The close association of these species with birds suggests that these vertebrates are the main source of food for these triatomines in the wild [16]. These vectors have already been found in bird nests of the families Dendrocolaptidae, Troglodytidae, Furnariidae and Icteridae [15,17,18,19,20,21,22,23].

The species of the genus *Psammolestes* form a monophyletic group and have the same chromosomal characteristics [24,25,26]. These vectors were initially grouped into a single tribe called Psammolestini as they presented morphological distinctions when compared to *Rhodnius* [27,28]. However, Lent and Wygodzinsky [16] considered this tribe as *nomen nudum* and, based on some morphological and ecological characters, they grouped it in the Rhodniini tribe [16,27,29]. In 2002 and more recently (2022), based on phylogenetic analyses, it was suggested to change the generic status of the three species of *Psammolestes* to the genus *Rhodnius* [30,31]. However, the generic status of *Psammolestes* was confirmed based on the biological species concept [32].

As a result of the importance of the correct identification of CD vectors, the taxonomic aspects of the *Psammolestes* spp. were revisited based on morphological and morphometric data.

## 2. Materials and Methods

### 2.1. Triatomines Examined

**Figure 1:**
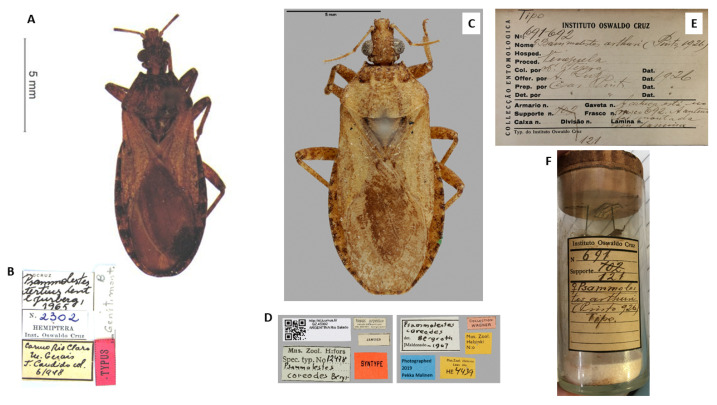
Types of *Psammolestes* species: (**A**) *P. tertius* holotype, (**B**) labels in detail (**C**) *P. coreodes* syntype, (**D**) labels in detail, (**E**) form with information on the type of *P. arthuri*, (**F**) the type of *P. arthuri* stored and sealed inside a glass tube.

*P. tertius* (deposited in the Collection of Triatomines of the Oswaldo Cruz Institute, Rio de Janeiro, Brazil) (Figure 1A,B), *P. coreodes* (deposited in the Zoological Museum, Helsinki, Finland; http://id.luomus.fi/GZ.45902, accessed on 14 October 2022, (Figure 1C,D) and *P. arthuri* (deposited in the Entomological Collection of the Oswaldo Cruz Institute, Rio de Janeiro, Brazil) (Figure 1E,F) were analyzed.

**Figure 2:**
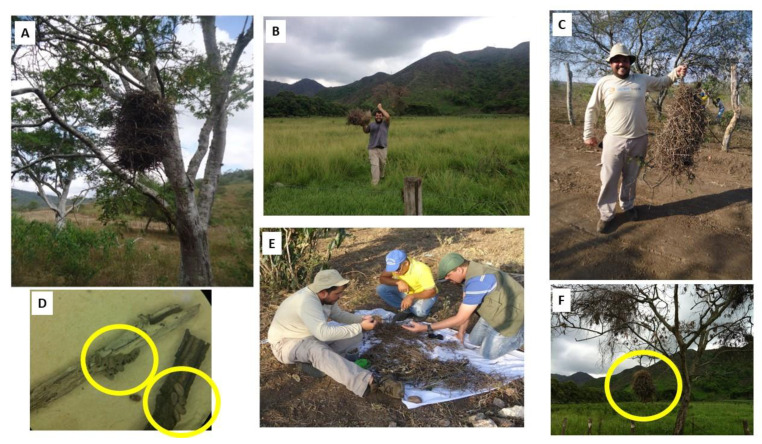
Aspects of the fieldwork. (**A**) A nest of *Phacellodomus* sp. in Brazil. (**B**) Nest collection in Venezuela. (**C**) Nest collection in Brazil. (**D**) Eggs adhering to nest sticks. (**E**) Nest sorting in the field. (**F**) *Phacellodomus* sp. in Venezuela.

In addition, field material was analyzed. For the collection of *Psammolestes* spp., fieldwork was carried out during the day in wild and rural environments, from specific points where nests of *Phacellodomus rufifrons* (Wied-Neuwied, 1821) (Passeriformes, Furnariidae) were observed (Figure 2A,F). These birds’ nests were removed from the trees with the help of a rope and were carefully placed on a white cloth to aid in visualizing the insects (Figure 2B,C,E). Subsequently, screening was carried out to separate the triatomines from the kindling. The triatomines were collected and organized in plastic bottles with filter paper and labeled with the data related to the point collected (Figure 2D,E). Information on the populations collected and used in the taxonomic studies is presented in Table 1.

### 2.2. Morphometric Studies

Observations were made using a stereoscopic microscope (EM) (Leica 205A) and measurements were taken using a MoticAdvanced 3.2 plus image analysis system. In addition, morphometric studies were also carried out on the eggs of the three species (from 50 eggshells of each species) and the mean, standard deviation, maximum and minimum for the width, length, area and diameter of the opercular opening were determined. All measurements were taken in millimeters (mm).

### 2.3. Morphological Studies

For morphological studies, we used 10 genitalia of each species; dissections of the male genitalia were performed by first removing the pygophore from the abdomen with forceps and then cleaning it in a 20% NaOH solution for 24 h. The dissected structures were studied and observations were made using an EM (Leica 205A) photographed in glycerol similar to as described by Lent and Jurberg [11].

For the scanning electron microscopy (SEM) analyses, three females and three males of *Psammolestes* spp. and five eggshells were cleaned in an ultrasound machine. Subsequently, the samples were dehydrated in alcohol, dried in an incubator at 45 °C for 20 min and fixed in small aluminum cylinders with a transparent glaze. Sputtering metallization was then performed on the samples for 2 min at 10 mA in an Edwards sputter coater. After this process, the samples were studied and photographed using SEM (JEOL, JSM-7500F), similar to as described by Rosa et al. [33]. The general morphological terminology used mainly follows Lent and Wygodzinsky [16]. The (visible) segments of labium were numbered II to IV, given that the first segment is lost or fused to the head capsule in Reduviidae [34]. The terminology of the male genitalia structures follows Lent and Wygodzinsky [16] and the female genitalia follows Rosa et al. [33].

All specimens used were deposited in the Dr. Jose Maria Soares Barata Triatominae Collection (CTJMSB) of the São Paulo State University “Julio de Mesquita Filho” (Unesp), School of Pharmaceutical Sciences (FCFAR), Araraquara, São Paulo, Brazil.

## 3. Results

### 3.1. Triatomine Collections

In November of 2015, 172 specimens of *P. tertius* were collected in the municipality of Castro Alves, Bahia, Brazil, and 26 in Seabra, Bahia, Brazil. In December 2015, 137 specimens of *P. coreodes* were collected in the municipality Corumbá, Mato Grosso do Sul, Brazil. Lastly, 40 specimens of *P. arthuri* were collected in March 2017 at the Central University of Venezuela, Maracay, Aragua, Venezuela. All detailed information is organized in Table 1.

### 3.2. Morphological Characters

#### 3.2.1. Male Genitalia (Redescription of the Male Genitalia)

##### *Psammolestes* *arthuri*

**Figure 3:**
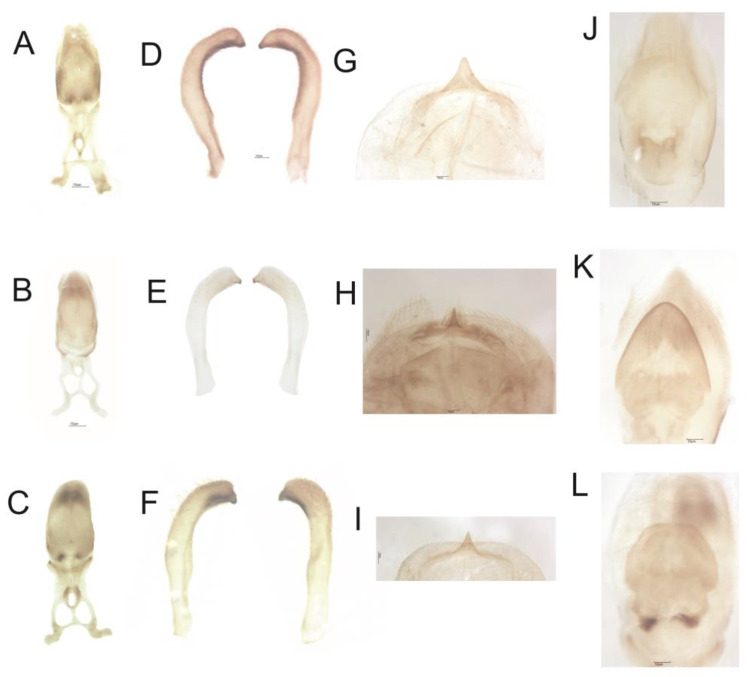
Structures of the diaphanized male genitalia. Phallosoma: (**A**) *P. arthuri*, (**B**) *P. coreodes* and (**C**) *P. tertius*. Parameters: (**D**) *P. arthuri*, (**E**) *P. coreodes* and (**F**) *P. tertius*. Median process of pygophore: (**G**) *P. arthuri*, (**H**) *P. coreodes* and (**I**) *P. tertius*. Phallosome plaque: (**J**) *P. arthuri*, (**K**) *P. coreodes* and (**L**) *P. tertius*.

The phallosoma (Ph) presents a quadrangular shape, the anterior borders are rounded and the (PrPh) has a small, rounded curvature; the extension of the basal plate (Eplb) is medium in size but broad in shape (Figure 3A). A cylindrical and hollow gonopore (PrG) process with internal edges joined at the base and at the apex, in which each extension consists of two parts, was inserted into the basal bridge by a narrow rod (Figure 3A). The phallosoma (Ph) plate was convex in shape (Figure 3J). The parameres (Pa) are of medium length, with cylindrical conformation, and in the side view are a little arcuate. In the general profile, they are sinuous with a pointed apex projected out of a side flap; the outer face has many sensilla; the inner face has sensilla and the protruding edges, where the end has a chitinized tip of different coloration from the body and this coloring marks the insertion of these sensilla (Figure 3D). A median pygophore process (PrP) is pointed with a blunt apex, and the end of the plate where it is inserted is poorly chitinized, located inside the outer edge of the pygophore (Figure 3G).

##### *Psammolestes* *coreodes*

The morphology of the phallosoma (Ph) had an ovoid shape, the anterior borders are acuminate and the (PrPh) has a small curvature; the extension of the basal plate (Eplb) is short but broad (Figure 3B). A cylindrical and hollow gonopore (PrG) process with internal edges joined at the base and at the apex, where each extension consists of two parts, was inserted into the basal bridge by a short rod (Figure 3B). The phallosoma plate shows an equilateral triangle shape with a rounded apex (Figure 3K). The parameres (Pa) are short, with cylindrical conformation through the lateral view, besides being slightly arcuate. In the general profile they are sinuous with a pointed apex projected coming out of a side flap; the outer face has many sensillae, the inner face has only a few and the tip is a different to the body (Figure 3E). The median process of the pygophore (PrP) is pointed with a very sharp apex, and the end of the plate where it is inserted has two strongly chitinized striae located inside the outer border of the pygophore (Figure 3H).

##### *Psammolestes* *tertius*

The phallosoma (Ph) has a rounded shape, the anterior edges are rounded and the (PrPh) has a small irregular curvature, and one has a long size (Figure 3C). (PrG) has inner edges joined at the base and at the apex, where each extension consists of two parts, and are inserted in the basal bridge by a narrow rod (Figure 3C). A has a rounded trapezoidal shape (Figure 3L). The parameres (Pa) are of medium length, with cylindrical conformation, and in the side view, a few are arched. In the general profile, they are sinuous with a pointed apex and projected coming out of a side flap; the outer face has many bristles, the inner face has fewer sensillae and the edges protrude to the point of having a chitinized tip of different coloring from the body (Figure 3F). The median process of the pygophore (PrP) is pointed with a sharp apex, and the end of the plate where it is inserted is little chitinized, situated inside the outer edge of the pygophore (Figure 3I).

### 3.3. Egg Morphometry

The mean, maximum and minimum deviation of the measurements of total length and eggshell length of populations of *P. arthuri*, *P. coreodes* and *P. tertius* are presented below in the form of a table (Table 2). 

When performing the intraspecific comparison, regarding significance, it was observed that they did not present any significance in any of the parameters used in the study. When performing the interspecific comparison, the species present a significant difference in the diameter of the opercular opening, but the total length is very significant (Table 3).

### 3.4. Morphological Studies

#### 3.4.1. Head

**Figure 4:**
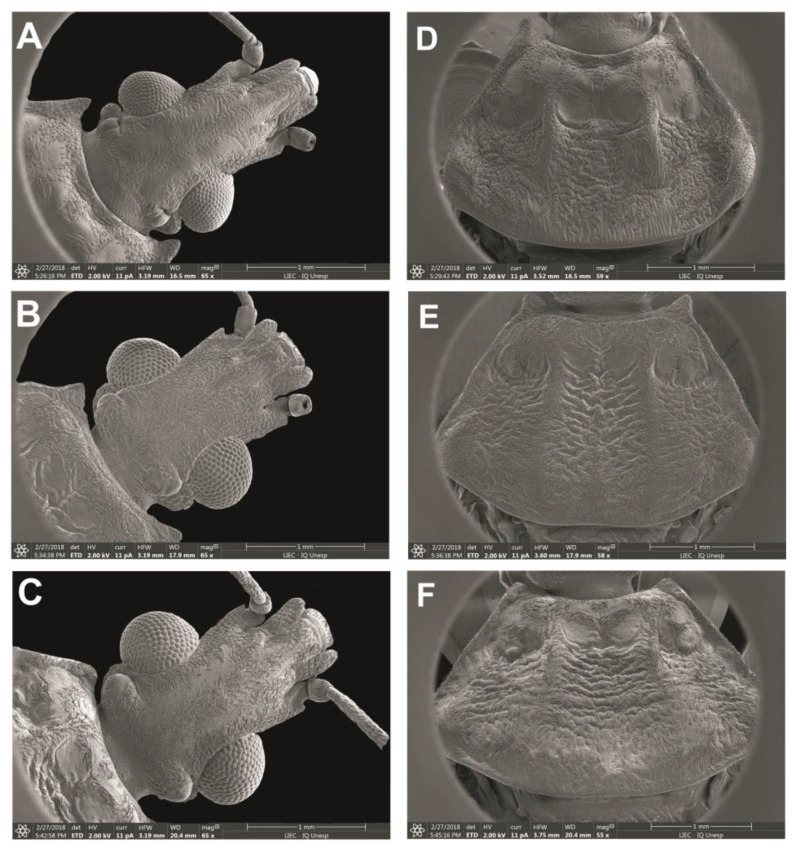
Dorsal view of head: (**A**) *P. arthuri*, (**B**) *P. coreodes* and (**C**) *P. tertius*. Pronotum: (**D**) *P. arthuri*, (**E**) *P. coreodes* and (**F**) *P. tertius*.

In *P. arthuri* (Figure 4A), the ocelli are separated and arranged in a straight line. In *P. coreodes* and *P. tertius* (Figure 4B,C), the ocellus form a symmetrical cylindrical shape and are larger in *P.tertius* when compared to *P.coreodes* and *P. arthuri*. The clypeus in *P. arthuri* is broad in the anterior portion and narrow in the posterior portion, with lateral lines transversely arranged (Figure 4A). In *P. coreodes*, it is rectangular in shape with straight lateral lines (Figure 4B) and in *P. tertius* it has a small narrowing in the anterior portion (Figure 4C). In *P. arthuri*, the anteclypeus is concave and narrow in the anterior portion and straight and wide in the posterior portion (Figure 4A). In *P. coreodes*, the anteclypeus is straight and broad in the anterior and posterior portions (Figsure 4B) and in *P. tertius*, the anteclypeus is concave and broad in the anterior portion and straight and wide in the posterior portion (Figure 4C).

#### 3.4.2. Thorax

**Figure 5:**
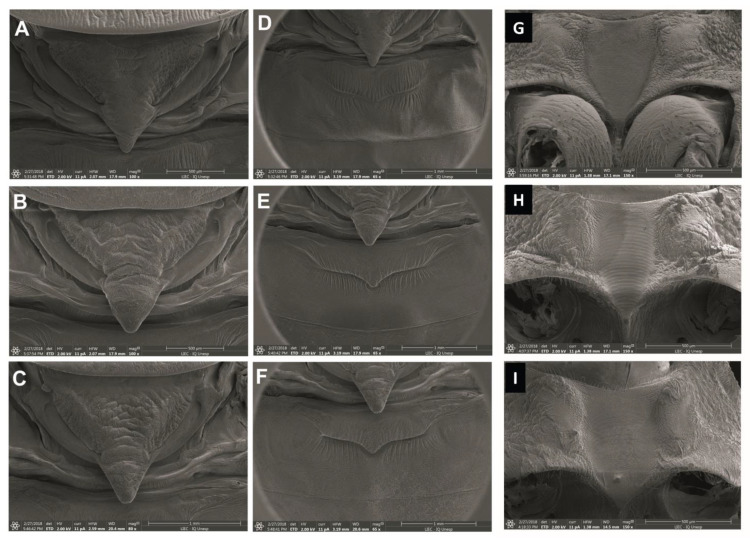
Scutellum: (**A**) *P. arthuri*, (**B**) *P. coreodes* and (**C**) *P. tertius*. Urotergite process I: (**D**) *P. arthuri*, (**E**) *P. coreodes* and (**F**) *P. tertius*. Stridulatory groove: (**G**). *P. arthuri*, (**H**). *P. coreodes* and (**I**). *P. tertius*.

Anterolateral angle: In *P. arthuri*, the insertion was performed near the border of the ocelli. The insertions are pronounced and the posterior extremities are triangular in shape (Figure 4D). In *P. coreodes*, the insertion was below the dividing line between the neck and the protorax; they are short, triangular and not very pronounced (Figure 4E). In *P. tertius*, the insertion was made below the dividing line between the neck and the protorax and are short and the ends are rounded (Figure 4F). In *P. arthuri*, the carinas are elevated from the anterior to the posterior lobe (Figure 4D). In *P. coreodes* and *P. tertius*, they are present but distributed with little prominence in the posterior lobe (Figure 4E,F). In *P. arthuri*, *P. coreodes* and *P. tertius*, the discal tubercles are not present (Figure 4D–F). The glabrous areas of all three species are distributed regularly in the central region of the pronotum and irregularly on the sides of the anterior lobe; in *P. arthuri* you can view six glabrous areas, *P. coreodes* presents another layout pattern with eight glabrous areas and *P. terius* has a pattern with twelve glabrous areas. The posterior lobe of *P. arthuri* is rough (Figure 4A) and in *P. coreodes* and *P. tertius* it is striated (Figure 4B,C). In *P. arthuri*, *P. coreodes* and *P. tertius*, the apical processes are extremely short; however, the tips of the apical processes are distinct due to the differences in the triangular conformation (Figure 5A–C). The central depression in *P. arthuri* is clearly delimited by two areas: a small, fluted area and a larger, flat area (Figure 5A). In *P. coreodes* and *P. tertius*, the delimitations are not accentuated and have irregular striations distributed throughout the region (Figure 5B,C).

In *P. arthuri*, *P. coreodes* and *P. tertius*, the limiting line of the estridulatory sulcus is slightly curved in the anterior portion (Figure 5G–I). In *P. arthuri*, the groove is short and broad throughout the groove body and shows a narrowing in the posterior portion (Figure 5G). In *P. coreodes*, the groove is short and broad in the anterior portion and presents two straight limiting lines in the medial and posterior “V” (Figure 5H). In *P. tertius*, the border is wide initially and narrows in the median region and the posterior portion is re-sharpened at the end of the posterior V-shaped portion (Figure 5I).

#### 3.4.3. Abdomen

In *P. arthuri*, the urotergite has a striated general appearance, but with vertical striae and ribs distributed throughout the upper and lower areas. Its apex is subtly rounded and does not extend beyond the central area (Figure 5D). *P. coreodes* presents a urotergite with a striated aspect vertically on the sides and lower region, but with discrete ridges and ribs in the upper region and marks in the lower region. Its apex is pointed and has a rounded marking in the central region (Figure 5E). *Psammolestes tertius* presents a urotergite with striations and discrete veins and its apex is pointed with a central depression (Figure 5F).

**Figure 6:**
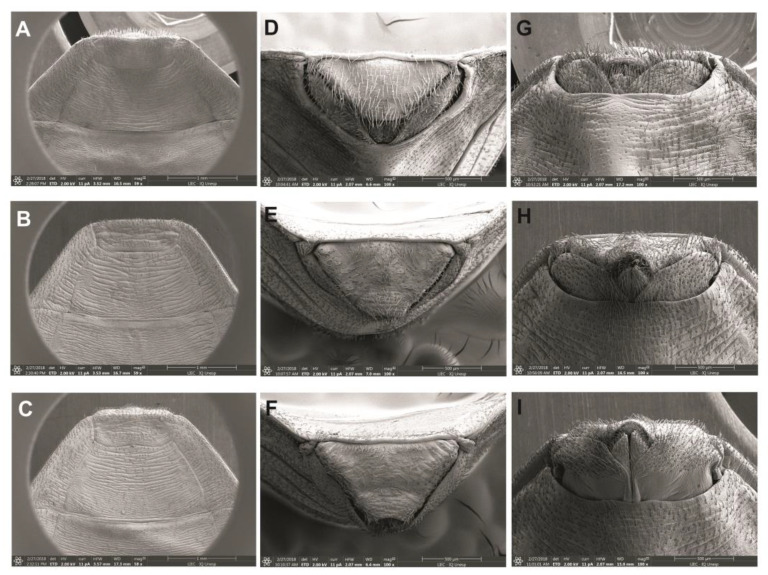
Female external genitalia using SEM. Dorsal view: (**A**) *P. arthuri*, (**B**) *P. coreodes* and (**C**) *P. tertius*. Posterior view: (**D**) *P. arthuri*, (**E**) *P. coreodes* and (**F**) *P. tertius*. Ventral view: (**G**) *P. arthuri*, (**H**) *P. coreodes* and (**I**) *P. tertius*.

Female external genitalia: dorsal view: The line dividing the seventh and eighth segments is concave in *P. Arthuri* (Figure 6A), straight transverse in *P. coreodes* (Figure 6B) and convex in the central portion in *P. tertius* (Figure 6C). The line dividing the eighth of the ninth segment is convex in *P. arthuri* (Figure 6A) and straight in *P. coreodes* and *P. tertius* (Figure 6B,C). The third segment of *P. arthuri* (Figure 6A) laterally presents as a pair of symmetric curves, differing from those of *P. coreodes* (Figure 6B) and *P. tertius* (Figure 6C), which are straight and diagonally arranged.

Posterior view: In *P. arthuri*, *P. coreodes* and *P. tertius* (Figure 6D–F), the ninth segment is flattened frontally, and is sharper in *P. tertius* (Figure 6F). The tenth segment in *P. arthuri*, *P. coreodes* and *P. tertius* (Figure 6D–F) is in the form of a “U” with more closed or more open contours. Gonocoxite 8 is visible in *P. arthuri* and not visible in *P. tertius* (Figure 6D,F). It has a peniform format, which varies in size, and is markedly higher in *P. arthuri* (Figure 6D). Gonopophysics 8 is visible in *P. arthuri*, is subtly visible in *P. tertius* (Figure 6D,F) and is not visible in *P. coreodes* (Figure 6E).

Ventral view: the dividing line of segment VII with gonocoxites 8 is concave and similar in *P. arthuri* and *P. coreodes* (Figure 6G,H) and differs in the central part in *P. tertius* (Figure 6I). Segment IX is irregular and has an undulating shape throughout its length in all three species (Figure 6G–I). The gonocoxite 8 in *P. arthuri* are rounded (Figure 6G), in *P. coreodes* they are convex in the lateral and border with gonapophysics 8 and are rounded in all other areas (Figure 6H) and in *P. tertius* they have a trapezoidal shape, gonapophysis 8 (Figure 6I). Gonapophysics 8 in *P. arthuri* (Figure 6G) is short and curved, while in *P. coreodes* and *P. tertius* they are triangular and similar (Figure 6H,I).

### 3.5. Dichotomous Key for Species of the Genus Psammolestes, Based on Adults

**Figure 7:**
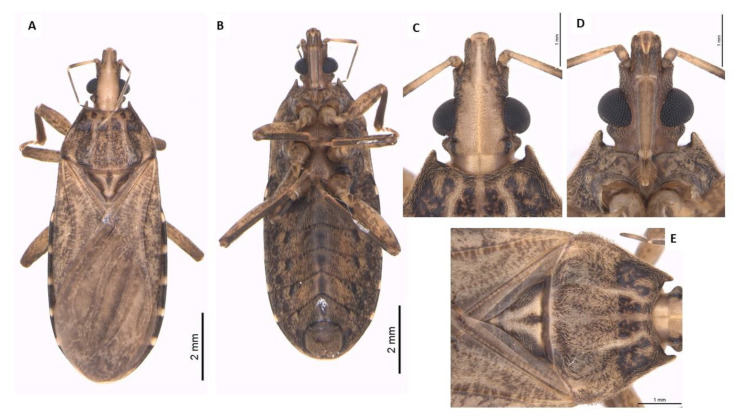
Adult male of *P. arthuri*. Dorsal view (**A**), ventral view (**B**), head dorsal view (**C**), head ventral view (**D**), thorax dorsal view (**E**).

Head and thorax highly polished; head not constricted before neck in lateral view (Figure 7); head has wide percurrent yellowish band occupying entire interocular space dorsally (Figure 7); long hairs on apex of second and third rostral segments (Figure 7); anterolateral angles of pronotum projecting forward to level of ocelli (Figure 7); male genitalia with large, fused basal plate struts…………………………………*P. arthuri* (Figure 7).

Head and thorax dull; head constricted before neck in side view; head light yellowish brown dorsally, speckled with darker coloration or dark and with narrow yellow percurrent line dorsally; long hairs on entire second and third rostral segments; shorter anterolateral angles, not attaining the level of ocelli; male genitalia with shorter, not fused basal plate struts ………………………………………………………………………………………2.

**Figure 8:**
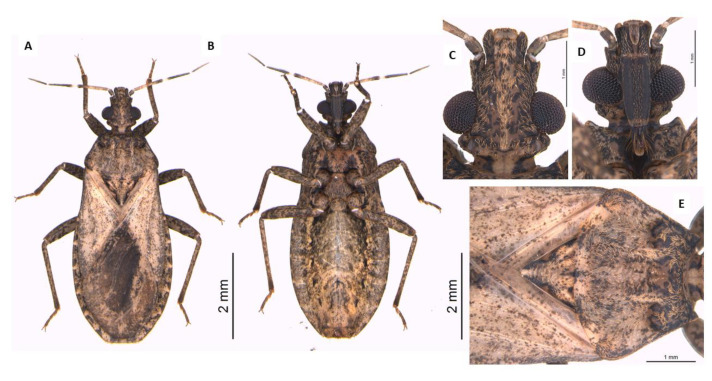
Adult female of *P. coreodes*. Dorsal view (**A**), ventral view (**B**), head dorsal view (**C**), head ventral view (**D**), thorax dorsal view (**E**).

2—Head as long as or slightly shorter than wide across eyes (Figure 8); anteocular region not over twice as long as postocular region (Figure 8); head strongly declivous behind ocelli (Figure 8); anterolateral angles of pronotum acuminate (Figure 8); male genitalia with very oval-shaped basal plate struts. Length of male: 11–13 mm and length of female: 12–14 mm. Width of pronotum of male: 3.0–3.5 and width of pronotum of female: 3.2–4.0 mm. Width of abdomen of male: 4–5 mm and width of abdomen of female: 4.5–5.5 mm. …………………………………………………………………………*P. coreodes* (Figure 8).

**Figure 9:**
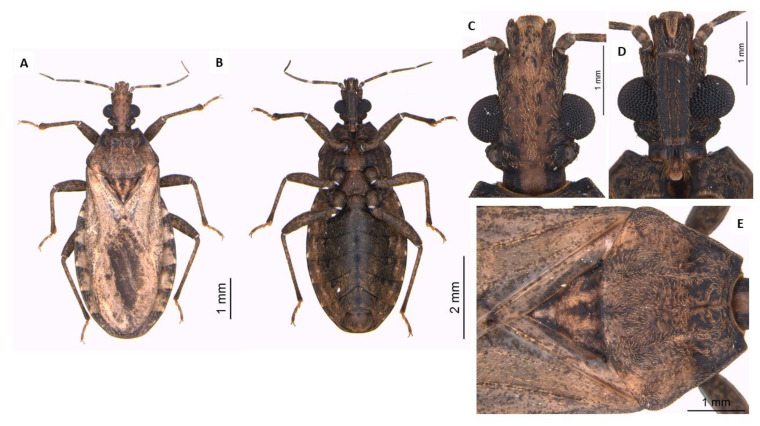
Adult male of *P. tertius*. Dorsal view (**A**), ventral view (**B**), head dorsal view (**C**), head ventral view (**D**), thorax dorsal view (**E**).

Head slightly longer than wide across eyes (Figure 9); anteocular region from two to two and one-half times as long as postocular (Figure 9); head moderately declivous behind ocelli (Figure 9); very short anterolateral angles of pronotum (Figure 9); male genitalia with broadly S-shaped basal plate struts; dorsal sclerotization of phallosoma is broadly rounded apically ……………………………………………………………*P. tertius*(Figure 9).

### 3.6. Morphological Description of Eggs

The eggs of *Psammolestes* spp. were studied. The eggs of this genus are strongly attached to the substrate and are of medium and small size.

#### 3.6.1. *Psammolestes arthuri*

**Figure 10:**
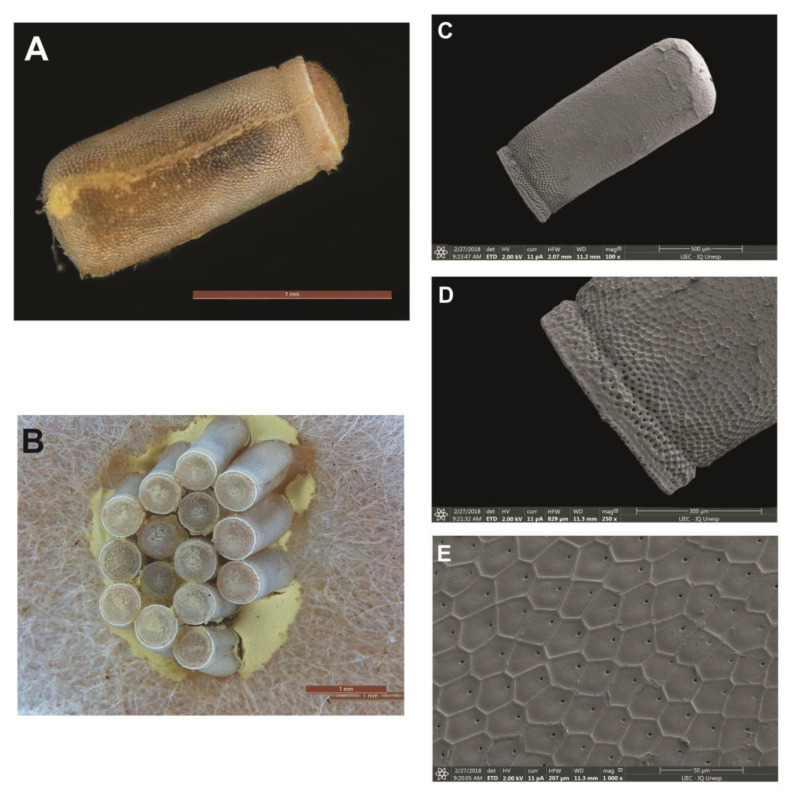
Eggs of *P. arthuri*. (**A**) Eggs by means of optical microscopy; (**B**) eggs adhered together in a substrate; (**C**) eggshells by means of scanning electron microscopy; (**D**) detail of the chorion border by means of SEM and (**E**) pattern of an exocorial cell by SEM.

The coloration ranged from off-white to brown, then approaching light brown when about to hatch. Eggs with continuous coloring have a small stripe on the chorion border lighter than the body of the egg. They do not have a “collar”, they have a long, narrow “collar”, often presenting a “lateral flattening” of coloration equal to the body of the egg. The operculum is light in color, in the same shade of the corial border which is a tone lighter than the body of the egg, with translucent and whitish edges. Its shape is circular with elevated, irregular filamentous projections and the opercular borders are narrow and of a light color; in some cases, there is a slight inclination of the operculum in relation to the “lateral flattening”. The eggs were significantly cylindrical, at an average of 1.54 mm in length (Table 2). The general aspect of the exocorial is not uniform, but the body is organized by people who are regular and the patients do not have access to the exocoriais cells. The pattern of the exocoriais cells is irregular and is not of a standardized smooth appearance. In optical microscopy (OM), by transparency, only the small central holes at the entrance of the tubes are evident. In SEM, the “boundary lines” (LL) are high and well visible in the presence of granulations (Figure 10A–E).

#### 3.6.2. *Psammolestes coreodes*

**Figure 11:**
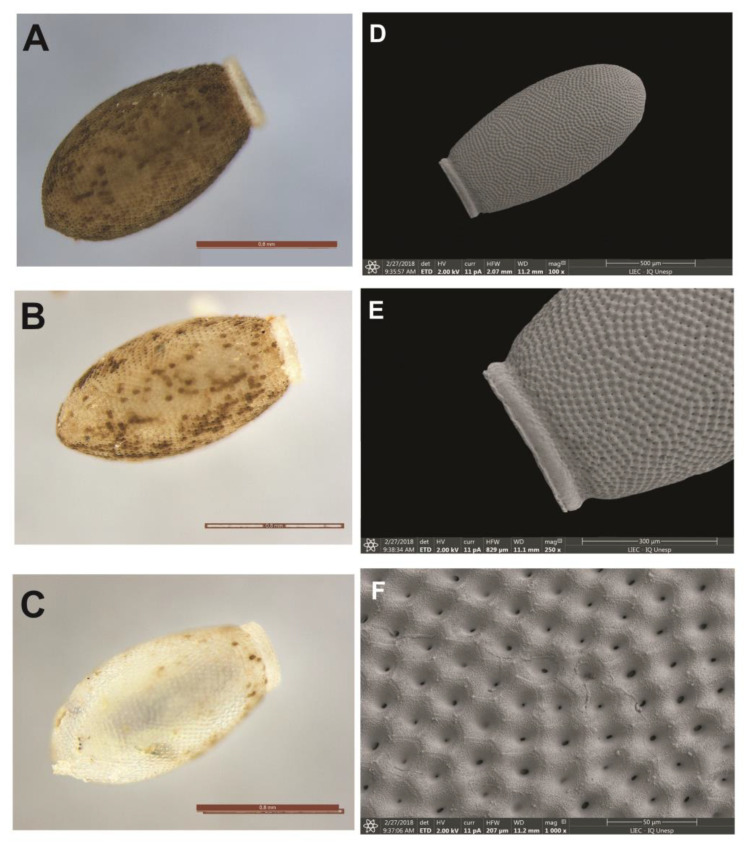
Eggs of *P. coreodes*. (**A**–**C**) Chromatic variations of eggs by means of light microscopy. (**D**) Eggshell by means of scanning electron microscopy. (**E**) Detail of the central border in SEM and (**F**) pattern of exocorial cells in SEM.

The coloration varies from whitish to brown, reaching light brown when about to hatch. They have a pronounced brown pigmentation pattern, with a specular appearance. Eggs have a double coloration, with one color clearly just below the “colo” and corial boards. They do not have a “collar”; they have a long, narrow “collar” and often have a “lateral flattening” of coloration equal to the body of the egg. The operculum is light in color, in the same shade as the corial border in a tone lighter than the body of the egg, with translucent and whitish edges. Its shape is circular with a subtle central projection and has no obvious opercular edges. There is a more evident pattern of pores in the central region. The eggs, markedly oval, have a mean diameter of 1.34 mm (Table 2). The general appearance of the exocore is uniform, in both the egg body and the operculum, and the polygonal areas vary from pentagonal to hexagonal, most of which are hexagonal. In OM, by transparency, only the small central holes at the entrance of the tubes are evident. In SEM, the “limiting lines” (LL), due to the presence of granulations, have a slightly rough appearance. By transparency, they are refringent and, consequently, not very evident. The granulations that cover the tegument are irregular in size, agglutinated and distributed throughout the area of the exocorial cell (Figure 11A–F).

#### 3.6.3. *Psammolestes tertius*

**Figure 12:**
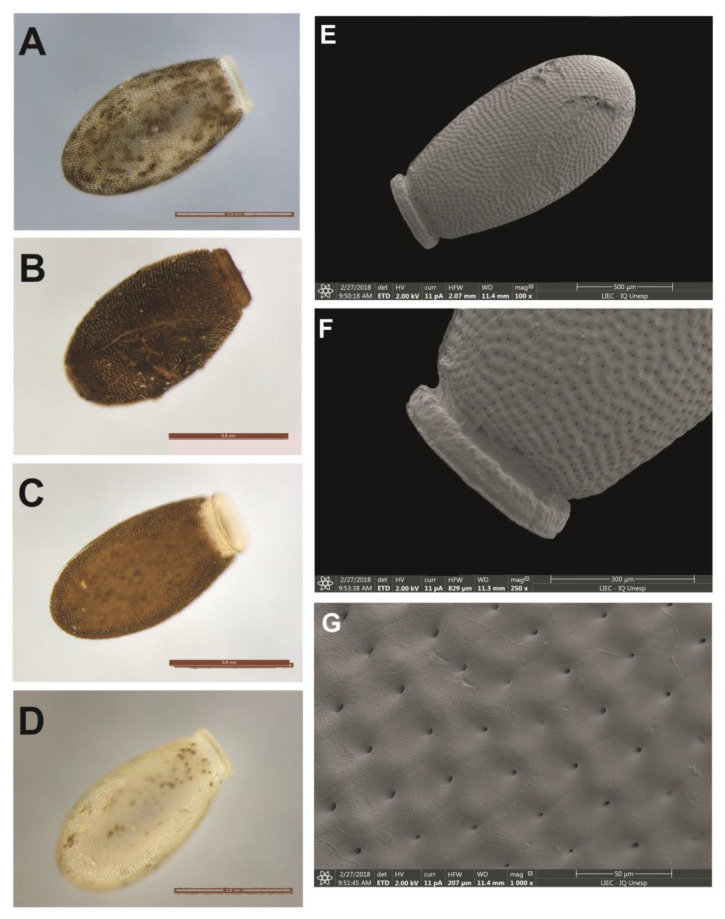
Eggs of *P. tertius*. (**A**–**D**) Chromatic patterns of eggs by means of optical microscopy; (**E**) eggshells by means of scanning electron microscopy; (**F**) detail of the chorion border by means of SEM and (**G**) pattern of exocorial cell in SEM.

The egg color has four patterns: one whitish with small spots of light brown color (Figure 12D), one with a gray color with larger brown stains (Figure 12A), one full brown (Figure 12B) and one light brown with a white collar and a corial lip (Figure 12C). A marginal pigmentation pattern determined a spotted appearance. They are “collared” and have a long, narrow “collar”, often showing a “lateral flattening” of coloration equal to the body of the egg. The operculum is light in color, in the same shade as the corial border in a tone lighter than the body of the egg, with translucent and whitish edges. Its shape is circular with a subtle central projection and has no obvious opercular edges. There is a more evident pattern of pores in the central region. The eggs, markedly oval, have an average diameter of 1.40 mm (Table 2). The general appearance of the exocoria is uniform, both in the egg body and in the operculum (the non-polygonal areas). In OM, by transparency, only the small central holes at the entrance of the tubes are evident. In SEM, the “boundary lines” (LL) are not present and cells have a slightly rough appearance. The granulations that cover the integument are of irregular size, agglutinated and distributed throughout the area of the exocorial cell (Figure 12A–G).

### 3.7. Dichotomous Key for Species of the Genus Psammolestes, Based on Eggs

           1. Long cylindrical shaped eggs with lateral flattening……                *P. arthuri*

                              2. Oval and circular shaped eggs……                                         3

          3a. Eggs of oval shape without the presence of the collar……            *P. coreodes*

         3b. Eggs in circular format with the presence of the collar……            *P. tertius*

## 4. Discussion

Although the integrative taxonomy shows a trend towards the taxonomy of Triatominae [5], the classical taxonomy based on morphological and morphometric characteristics is of great importance for the correct classification of species (mainly because the diagnosis of practically all triatomine species is based on morphological data) [5]. This methodology was used to describe the species of *Psammolestes* [8,9,11], and was the process used to characterize pygophore described by Lent and Jurberg [11]. They used the marked character to separate the three species.

In 1965, Lent and Jurberg [11] conducted a review of the genus *Psammolestes*, in which they described the species *P. tertius*. In this work, a brief redescription of the three species was given, describing the following characteristics: median process of pygophore, parameres and median extension of the basal plaque and phallosoma. Furthermore, the redescription of male internal genitalia was performed, with the following details: phallosoma, gonopore process, phallosoma plate, parameres and median process of the pygophore.

Based on this information, Lent and Wygodzisky [16] proposed a dichotomous key for the genus *Psammolestes*. Although alternative keys have been recently proposed to assist in the correct identification of triatomines (such as the keys proposed from cytogenetic data [35,36,37,38]), the dichotomous keys based on morphological characteristics are the most commonly used from a practical scientific point of view. Thus, we enriched the Lent and Wygodzisky [16] key with new characters to assist in the correct identification of the species within this genus.

In 1976, Carcavallo and Tonn [39] developed a dichotomous key to differentiate some triatomine genera from egg character; however, they did not include *Psammolestes* spp. Subsequently, Barata [40] and Santos et al. [41] presented specific keys for the genus *Rhodnius*. In 1975, Carcavallo et al. [42] published the first characteristics of *Psammolestes* eggs, with an emphasis on *P. arthuri*. Barata [43] succinctly described the eggs of the genus *Psammolestes*. Thus, the key proposed in the present study for *Psammolestes*, together with the keys of Barata (1981) and Santos et al. (2009), allow species of the Rhodniini tribe to be identified from eggs.

Recent phylogenetic studies demonstrate that *Psammolestes* species are phylogenetically related, with *P. tertius* and *P. coreodes* being sister species [25]. In addition, one of the models tested for species delimitation (the ‘deep divergence and large population size’ model) delimited only two species: (i) *P. arthuri* and (ii) *P. coreodes* + *P. tertius* [25]. However, reproductive barriers confirm the specific status of *P. coreodes* and *P. tertius* based on the biological species concept [44]. Furthermore, the morphological study conducted by SEM allowed to differentiate the three species of *Psammolestes*; in the dorsal portion of the thorax, differences were noted in the pronotum, anterior lateral angles, the sub median carinas, glabrous areas and the scutellum and in the ventral portion of the thorax, differences in the shape of the stridulatory sulcus were observed. The female external genitalia were examined and differences were observed when examined by the dorsal, posterior and ventral view.

Hypsa et al. [30] and Filée et al. [31] emphasize that within the Rhodniini, it is evident that *Psammolestes* spp. represent species of *Rhodnius*. However, the characteristics of the head described by SEM (arrangement and size of the ocelli and the shape of the clypeus and the anteclypeus) when compared to *Rhodnius* do not confirm the inclusion of *Psammolestes* in the genus *Rhodnius*. These results based on the phenetic species concept are in agreement with the recent confirmation of generic status proposed based on the biological species concept [32].

## 5. Conclusions

Based on this, it was possible to differentiate between three species of *Psammolestes* and confirm that this genus should not be classified in the genus *Rhodnius*, contributing to Rhodniini’s taxonomy. Overall, our morphological data will be clear and helpful for a field survey of Chagas disease in the near future.

## Figures and Tables

**Table 1:** Collection data of *Psammolestes* spp.

Species	Country	State	Municipality	Locality
*P. tertius*	Brazil	Bahia	Seabra	Agreste
*P. tertius*	Brazil	Bahia	Castro Alves	Melancia II
*P. coreodes*	Brazil	Mato Grosso do Sul	Corumbá	Access road to “Que Qué”
*P. coreodes*	Brazil	Mato Grosso do Sul	Corumbá	Access road to Paraguai River
*P. tertius*	Brazil	Mato Grosso do Sul	Corumbá	Access road to Alegria farm
*P. arthuri*	Venezuela	Aragua	Maracay	Central University of Venezuela

**Table 2:** Results of the measurements and respective statistical analyses of the opercular opening and the total length of 50 eggshells of populations of three species of *Psammolestes*.

	Opercular Opening				Total Length			
Population	Minimum	Maximum	Average	Standard Deviation	Minimum	Maximum	Average	Standard Deviation
*P. tertius* POP1	0.30	0.37	0.34	0.017	1.27	1.47	1.36	0.052
*P. tertius* POP2	0.30	0.38	0.35	0.02	1.32	1.5	1.4	0.046
*P. coreodes* POP3	0.29	0.35	0.32	0.016	1.25	1.43	1.34	0.05
*P. coreodes* POP4	0.29	0.37	0.32	0.018	1.26	1.43	1.34	0.046
*P. coreodes* POP5	0.26	0.36	0.31	0.022	1.2	1.46	1.33	0.051
*P. coreodes* POP6	0.28	0.34	0.31	0.015	1.25	1.44	1.36	0.051
*P. arthuri* POP7	0.39	0.47	0.43	0.02	1.34	1.71	1.54	0.085

POP1: Castro Alves—BA; POP2: Santa Therezinha—BA; POP3: (Que Que) Corumbá—MS; POP4: (Alegria farm access) Corumbá—MS; POP5: (Paraguai River acess) Corumbá—MS; POP6: (Access to morro of Urucum) Corumbá—MS and POP7: Maracay, Aragua–Venezuela.

**Table 3:** Statistical analysis using unpaired t-test with Welch’s correction for measurements of the opercular opening and total length of 50 eggshells of the genus *Psammolestes* populations.

	Opercular Opening		Total Length	
Populations	*p*-Value	Significance	*p*-Value	Significance
POP1 vs. POP2	*p* > 0.05	NS	*p* > 0.05	NS
POP1 vs. POP3	*p* > 0.05	NS	*p* > 0.05	NS
POP1 vs. POP4	*p* > 0.05	NS	*p* > 0.05	NS
POP1 vs. POP5	*p* > 0.05	NS	*p* > 0.05	NS
POP1 vs. POP6	*p* > 0.05	NS	*p* > 0.05	NS
POP2 vs. POP3	*p* > 0.05	NS	*p* < 0.05	*
POP2 vs. POP4	*p* > 0.05	NS	*p* < 0.05	*
POP2 vs. POP5	*p* > 0.05	NS	*p* < 0.05	*
POP2 vs. POP6	*p* > 0.05	NS	*p* > 0.05	NS
POP3 vs. POP4	*p* > 0.05	NS	*p* > 0.05	NS
POP3 vs. POP5	*p* > 0.05	NS	*p* > 0.05	NS
POP3 vs. POP6	*p* > 0.05	NS	*p* > 0.05	NS
POP7 vs. POP1	*p* > 0.05	NS	*p* < 0.01	**
POP7 vs. POP2	*p* > 0.05	NS	*p* < 0.01	**
POP7 vs. POP3	*p* < 0.05	*	*p* < 0.01	**
POP7 vs. POP4	*p* < 0.05	*	*p* < 0.01	**
POP7 vs. POP5	*p* < 0.05	*	*p* < 0.01	**
POP7 vs. POP6	*p* < 0.05	*	*p* < 0.01	**

POP1: *P. tertius*: Castro Alves—BA; POP2: *P. tertius*: Santa Therezinha—BA; POP3: *P. coreodes* (Que Que) Corumbá—MS; POP4: *P. coreodes* (Alegria farm acess) Corumbá—MS; POP5: *P. coreodes* (Paraguai River acess) Corumbá—MS; POP6: *P. coreodes* (Acess to morro do Urucum) Corumbá—MS and POP7: *P. arthuri* Maracay, Aragua—Venezuela. NS = not significant (*p* > 0.05); * significant (*p* < 0.05); ** very significant (*p* < 0.01).

## Data Availability

All relevant data are within the manuscript.
